# The burden of cervical cancer in China

**DOI:** 10.3389/fonc.2022.979809

**Published:** 2022-09-20

**Authors:** Xiu Shen, Yiquan Cheng, Fupeng Ren, Zhilong Shi

**Affiliations:** Department of Hematology and Oncology, Hwa Mei Hospital, University of Chinese Academy of Sciences, Ningbo, China

**Keywords:** cervical cancer, trends, incidence, disability-adjusted life-years (DALYs), mortality

## Abstract

**Objective:**

Trends in the incidence, disability-adjusted life-years (DALYs), and mortality rate of cervical cancer remain unknown.

**Methods:**

The average annual percent changes (AAPCs) and relative risks (RR) in the incidence, DALYs, and mortality rate were determined using a joinpoint regression analysis; the net age, period, and cohort effects on above rates were evaluated.

**Results:**

A significant increase in age-standardized incidence (AAPC, 0.9%; 95CI: 0.8, 1.1) but significant decreases in age-standardized DALYs (AAPC, -0.4%; 95%CI: -0.60, -0.20) and the mortality rate (AAPC, -0.4%; 95CI: -0.6, –0.3) were observed. As for age-specific rates, the incidence was higher in younger age groups, and the DALYs and mortality rate were lower in older age groups. The effects of age included a slight but significant increase in the RR with advancing age from 35 to 94 years; the period effect included a significant increase in the incidence over the 2005–2019 periods; and the cohort effect included a substantial increase in the incidence from earlier to later birth cohorts.

**Conclusions:**

The incidence of cervical cancer increased from 1990 to 2019, particularly in younger age groups, and the DALYs and mortality rate decreased in the older age groups. Furthermore, the incidence increased with age, period, and cohort.

## Introduction

Cervical cancer is among the most common gynecological malignancies in women (1–3). Approximately 570 000 new cases of cervical cancer and 311 000 deaths occurred in 2018, and China is among the countries with the greatest cervical cancer burden (4).

Most age-period-cohort (APC) studies of cervical cancer have focused mainly on the mortality rate (5, 6). However, great changes have taken place in aging and lifestyle in China, which may have great influence on the cervical cancer incidence and mortality rates. In addition, the trends in cervical cancer disability-adjusted life-years (DALYs) in China remain unclear. The APC effect on cervical cancer incidence and DALYs has not been fully investigated. Hence, it is of vital importance to estimate the time trends and APC effect on the cervical cancer incidence, DALYs, and mortality rate in China from 1990 to 2019.

To better understand the long-term trends in the cervical cancer burden, an APC model was used to investigate possible reasons for the temporal trend. This study sought to investigate long-term trends in the incidence, DALYs, and mortality rate of cervical cancer in China using data from the Global Burden of Diseases, Injuries, and Risk Factors Study 2019 (GBD 2019) and to explore the effects of age, period, and cohort independently under the APC framework.

## Materials and methods

We used data from the GBD 2019, which reported the comparative assessment of health loss and associated risk factors for 363 diseases and injuries in 204 countries and territories from 1990 to 2019 (7). Detailed descriptions of the method and approach of the GBD 2019 have been published elsewhere (7). The GBD study has been performed in accordance with the Guidelines for Accurate and Transparent Health Estimates Reporting. Hwa Mei Hospital, University of Chinese Academy of Sciences reviewed and approved this study.

The Bayesian meta-regression tool DisMod-MR 2.1 was the main method used for estimation. DisMod-MR 2.1 is a meta-analysis tool that uses a compartmental model structure with a series of differential equations that synthesize sparse and heterogeneous epidemiological data (7). Causes of death due to cervical cancer are modeled using the Cause of Death Ensemble model (CODEm) (7). All estimates are reported in terms of age-standardized rates per 100,000 persons. Population estimates independently produced by GBD 2019 were used as references for the calculation of age-standardized death rates and age-standardized DALYs. We used the GBD world population to calculate the age-standardized rates. The data are reported as estimates with 95% uncertainty intervals (UIs).

Data related to the cervical cancer disease burden were obtained from the Global Health Data Exchange database. The original data used by the GBD to estimate the incidence and mortality rate of cervical cancer in China were mainly obtained from the Cause of Death Reporting System of the Chinese Centers for Disease Control and Prevention (CDC), Disease Surveillance Points (DSPs), and the Maternal and Child Surveillance System.

To determine the magnitude of the secular trends, the average annual percent changes (AAPCs) and corresponding 95% confidence intervals (CIs) were evaluated using a joinpoint regression analysis (8). The AAPC was calculated as a geometrically weighted average of various annual percent change values from joinpoint regression analysis (9). The joinpoint analysis was performed using Joinpoint software obtained from the Surveillance Research Program of the U.S. National Cancer Institute.

The Joinpoint regression model equation is as follows:


$$\text{E}\left[\text{y}|\text{x}\right]\;=\;{\text{β}}_{0}\;+\;{\text{β}}_{1}\text{x }+\;{\text{δ}}_{1}\;{\left(\text{x }-\;{\text{τ}}_{1}\right)}^{+}\;+\;\ldots \;+\;{\text{δ}}_{\text{k}}\;{\left(\text{x }-\;{\text{τ}}_{\text{k}}\right)}^{+}$$

y refers to rates; x refers to the year of investigation; β refers to the constant term in the model; δi (i =1,2,… k) refers to the coefficient of the piecewise function; τi (=1,2,… k) refers to an unknown inflection point, also known as a turning point; a+ refers to when a > 0, a += a, otherwise a = 0.

In an APC model with the intrinsic estimato(IE) method, the age-specific rates were appropriately recoded into successive 5-year age groups (20–24, 25–29, …, 80–84), consecutive 5-year periods from 1990 to 2019, and consecutive 5-year birth cohort groups (1898-1902, 1903-1907, …, 1988-1992, 1993-1997), which were used to estimate the net age, time period, and cohort effects on the cervical cancer incidence, DALYs, and mortality rate (10, 11). The groups younger than 15 years and older than 80 years of age were excluded from this model. The APC model can be expressed as:


Y = log(M) = µ + ;α * age * i + β * period*j + γ * cohort * k + ϵ.

M represents the incidence, DALYs, and mortality rate, while α, β, and γ refer to the coefficients of three parameters: α refers to the age effect, which is the risk of three parameters in a particular age group; β is the period effect, which is the risk of three parameters in a given period; and γ is the cohort effect, which is the risk of three parameters for all people in the same birth cohort. µ and ϵ are defined as the intercept and random error, respectively (10, 11). The APC model was analyzed using Stata 18.0 software (Stata Corp, College Station, TX, USA).

## Results

**Figure 1:**
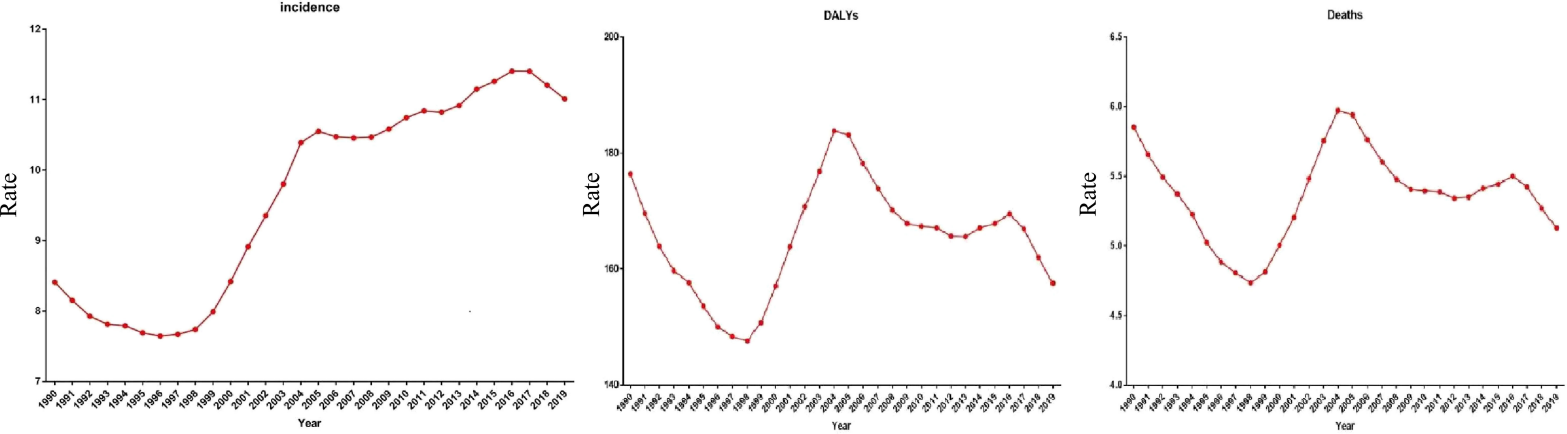
Trends of the incidence, disability-adjusted life-years (DALYs), mortality for cervical cancer 1990-2019, at all ages.

**Figure 1** shows the trends in the age-standardized incidence, DALYs, and mortality rate of cervical cancer in the Chinese population from 1990 to 2019. The age-standardized incidence declined from 1990 to 1998 and increased thereafter until 2019. The DALYs and mortality rate decreased from 1990 to 1998, increased until 2004, and then decreased thereafter with fluctuations. From 1990 to 2019, the age-standardized incidence increased from 8.41 to 11.00 per 100,000 persons, the age-standardized DALYs declined slowly from 176.39 to 157.50 per 100,000 persons, and the age-standardized mortality rate declined slowly from 5.85 to 5.12 per 100,000 persons.

**Table 1** shows the AAPCs of the cervical cancer incidence, DALYs and mortality rate from 1990 to 2019. During this period, the age-standardized incidence increased significantly (AAPC = 0.9%; 95% CI = 0.8 to 1.1), but the age-standardized mortality rate (AAPC = -0.4%; 95% CI = -0.6 to –0.3) and age-standardized DALYs decreased significantly (AAPC = -0.4%; 95% CI = -0.60 to -0.20). From 1998 to 2014, both the age-standardized incidence (AAPC = 5.3%; 95% CI = 4.9 to 5.6) and the age-standardized mortality rate increased significantly (AAPC = 4.3%; 95% CI = 3.9 to 4.8), while the age-standardized DALYs decreased significantly (AAPC = 4.1%; 95% CI = 3.70 to 4.40). Regarding age-specific rates, the incidence increased in the younger age groups (20–24, 30–34, 35–39, 40–44, 45–49, and 50–54 years), while the DALYs and mortality rate decreased in the older age groups (65–69, 70–74, 80–84, 85–89 years) (**Table 2**).

**Table 1:** Trends in cervical cancer incidence, mortality and DALY rates in China, 1990–2019.

Segments	Year	APC * (95% CI)
trend1	1990-1998	-2.8* (-3.0, -2.6)
trend2	1998-2004	4.3* (3.9, 4.8)
trend3	2004-2009	-2.1* (-2.7, -1.6)
trend4	2009-2017	0.2 (-0.1, 0.4)
trend5	2017-2019	-3.0* (-4.7, -1.2)
AAPC*	1990-2019	-0.4* (-0.6, -0.3)
trend1	1990-1993	-2.5* (-3.2, -1.7)
trend2	1993-1998	-0.4 (-0.8, 0.1)
trend3	1998-2004	5.3* (4.9, 5.6)
trend4	2004-2008	0.2 (-0.5, 1.0)
trend5	2008-2017	1.0* (0.8, 1.1)
trend6	2017-2019	-1.9* (-3.4, -0.4)
AAPC*	1990-2019	0.9* (0.8, 1.1)
trend1	1990-1992	-3.8* (-5.40, -2.20)
trend2	1992-1998	-1.9* (-2.20, -1.50)
trend3	1998-2004	4.1* (3.70, 4.40)
trend4	2004-2009	-2.0* (-2.50, -1.50)
trend5	2009-2017	0.00 (-0.20, 0.20)
trend6	2017-2019	-3.1* (-4.60, -1.50)
AAPC*	1990-2019	-0.4* (-0.60, -0.20)

DALYs, Disability-adjusted life-years; APC, annual percentage change; AAPC, average annual percent change. * Indicates statistical significance.

**Table 2:** The average annual percent changes (AAPCs) in age-specific incidence, mortality and DALY rates of cervical cancer in China, 1990–2019.

Age Group	Incidence (AAPC)	Mortality (AAPC)	DALY (AAPC)
Age-standardized rate	0.9* (0.8, 1.1)	-0.4* (-0.6, -0.3)	-0.4* (-0.6, -0.2)
20-24	0.9* (0.20, 1.60)	-1.4* (-2.2, -0.7)	-1.3* (-2.1, -0.6)
25-29	1.10 (-0.70, 2.90)	-1.2 (-2.9, 0.6)	-1.1 (-2.8, 0.7)
30-34	1.6* (0.60, 2.60)	-0.7 (-2.1, 0.8)	-0.5 (-2.0, 0.9)
35-39	2.0* (1.50, 2.60)	-0.2 (-0.8, 0.4)	-0.1 (-0.7, 0.5)
40-44	2.0* (1.50, 2.60)	-0.1 (-0.5, 0.4)	0.0 (-0.4, 0.5)
45-49	1.4* (0.70, 2.10)	-0.4 (-1.1, 0.4)	-0.3 (-1.0, 0.4)
50-54	1.3* (0.90, 1.60)	-0.1 (-0.6, 0.4)	-0.1 (-0.5, 0.4)
55-59	0.80 (-0.50, 2.10)	-0.4 (-1.0, 0.3)	-0.4 (-1.0, 0.3)
60-64	0.40 (-0.30, 1.10)	-0.5 (-1.2, 0.1)	-0.5 (-1.2, 0.1)
65-69	0.10 (-0.50, 0.70)	-0.5* (-1.0, -0.1)	-0.5* (-1.0, -0.1)
70-74	-0.30 (-0.50, 0.00)	-0.7* (-0.9, -0.4)	-0.7* (-0.9, -0.4)
75-79	-0.30 (-0.90, 0.40)	-0.6 (-1.2, 0.1)	-0.6 (-1.2, 0.1)
80-84	-0.30 (-0.80, 0.20)	-0.5 (-1.0, 0.0)	-0.5* (-1.0, 0.0)
85-89	-0.4* (-0.80, -0.10)	-0.5* (-0.8, -0.2)	-0.5* (-0.9, -0.2)
90-94	0.10 (-0.20, 0.40)	0.1 (-0.2, 0.4)	0.0 (-0.3, 0.3)

DALYs, Disability-adjusted life-years. *indicates statistical significance.

**Figure 2:**
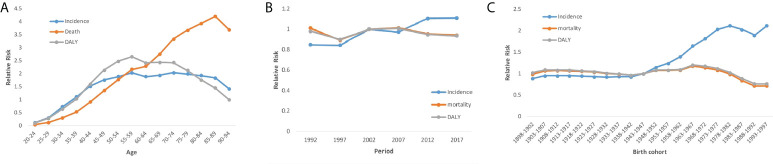
Cervical cancer incidence, disability-adjusted life-years (DALYs), mortality relative risks due to **(A)** age; **(B)** period; and **(C)** cohort effects.

After controlling for period and cohort effects, the analysis of the age effect on cervical cancer incidence showed that the relative risk (RR) increased significantly by 28.18 times with advancing age from 35 to 94 years (35–39 to 90–94 years; **Figure 2**) (**Table 3**). These two values were computed by their own coefficients (**Table 4**). In summary, the RR of mortality increased significantly with advancing age.

**Table 3:** Cervical cancer incidence, mortality and DALY relative risks (RR) due to age, period, and cohort effects.

Factor	Incidence (RR, 95% CI)	Mortality (RR, 95% CI)	DALY (RR, 95% CI)
**Age**			
20-24	0.11 (0.10,0.12)	0.04 (0.03,0.04)	0.10 (0.09,0.11)
25-29	0.30 (0.28,0.31)	0.12 (0.11,0.13)	0.28 (0.26,0.29)
30-34	0.72 (0.70,0.75)	0.29 (0.28,0.31)	0.63 (0.61,0.66)
35-39	1.10 (1.06,1.14)	0.52 (0.50,0.55)	1.02 (0.99,1.06)
40-44	1.52 (1.47,1.56)	0.90 (0.87,0.93)	1.59 (1.55,1.64)
45-49	1.76 (1.71,1.80)	1.35 (1.31,1.39)	2.13 (2.07,2.18)
50-54	1.89 (1.84,1.94)	1.76 (1.71,1.81)	2.47 (2.41,2.53)
55-59	2.03 (1.98,2.08)	2.16 (2.10,2.21)	2.65 (2.59,2.71)
60-64	1.88 (1.83,1.93)	2.28 (2.22,2.34)	2.41 (2.35,2.46)
65-69	1.93 (1.88,1.98)	2.74 (2.67,2.80)	2.42 (2.37,2.48)
70-74	2.03 (1.98,2.08)	3.33 (3.25,3.41)	2.42 (2.37,2.48)
75-79	1.98 (1.93,2.03)	3.67 (3.58,3.75)	2.12 (2.07,2.17)
80-84	1.92 (1.87,1.97)	3.93 (3.84,4.02)	1.75 (1.71,1.80)
85-89	1.83 (1.79,1.88)	4.20 (4.10,4.30)	1.44 (1.40,1.48)
90-94	1.41 (1.37,1.45)	3.69 (3.60,3.78)	0.99 (0.96,1.02)
**Period**			
1990-1994	0.85 (0.80,0.90)	1.01 (0.96,1.06)	0.98 (0.92,1.04)
1995-1999	0.84 (0.80,0.89)	0.89 (0.85,0.94)	0.90 (0.85,0.95)
2000-2004	1.00 (1.00,1.00)	1.00 (1.00,1.00)	1.00 (1.00,1.00)
2005-2009	0.97 (0.93,1.02)	1.01 (0.97,1.06)	1.01 (0.96,1.06)
2010-2014	1.10 (1.05,1.16)	0.95 (0.91,1.00)	0.95 (0.90,1.00)
2015-2019	1.11 (1.05,1.17)	0.94 (0.90,0.98)	0.93 (0.88,0.99)
**Cohort**			
1898-1902	0.89 (0.28,2.83)	0.99 (0.52,1.88)	1.02 (0.20,5.21)
1903-1907	0.95 (0.61,1.48)	1.06 (0.82,1.38)	1.09 (0.60,1.97)
1908-1912	0.95 (0.74,1.23)	1.07 (0.91,1.25)	1.09 (0.79,1.49)
1913-1917	0.95 (0.80,1.13)	1.06 (0.95,1.19)	1.08 (0.89,1.31)
1918-1922	0.94 (0.83,1.07)	1.05 (0.96,1.15)	1.06 (0.93,1.22)
1923-1927	0.93 (0.84,1.03)	1.04 (0.96,1.12)	1.04 (0.94,1.17)
1928-1932	0.92 (0.84,1.00)	1.01 (0.94,1.08)	1.01 (0.92,1.11)
1933-1937	0.93 (0.86,1.01)	0.99 (0.93,1.05)	0.99 (0.92,1.08)
1938-1942	0.93 (0.86,1.00)	0.96 (0.91,1.02)	0.96 (0.89,1.04)
1943-1947	1.00 (1.00,1.00)	1.00 (1.00,1.00)	1.00 (1.00,1.00)
1948-1952	1.14 (1.07,1.22)	1.08 (1.02,1.14)	1.08 (1.02,1.16)
1953-1957	1.24 (1.16,1.33)	1.08 (1.02,1.14)	1.08 (1.01,1.16)
1958-1962	1.40 (1.30,1.50)	1.08 (1.02,1.15)	1.09 (1.02,1.17)
1963-1967	1.65 (1.53,1.77)	1.18 (1.10,1.26)	1.20 (1.12,1.29)
1968-1972	1.81 (1.68,1.96)	1.13 (1.05,1.23)	1.17 (1.08,1.27)
1973-1977	2.03 (1.86,2.22)	1.08 (0.98,1.20)	1.12 (1.02,1.24)
1978-1982	2.12 (1.91,2.35)	0.99 (0.86,1.13)	1.03 (0.90,1.16)
1983-1987	2.02 (1.78,2.30)	0.84 (0.70,1.01)	0.88 (0.75,1.04)
1988-1992	1.89 (1.57,2.29)	0.72 (0.53,0.97)	0.76 (0.59,0.98)
1993-1997	2.12 (1.45,3.10)	0.71 (0.37,1.39)	0.76 (0.45,1.30)

**Table 4:** Age-period-cohort (APC) model analysis results of cervical cancer incidence, mortality and DALY rates in China.

Variable	Incidence (Coef, 95% CI)	Mortality (Coef, 95% CI)	DALY (Coef, 95% CI)
**Age**			
20-24	-0.66 (-0.75, -0.57)	-0.03 (-0.12,0.06)	-0.07 (-0.17,0.03)
25-29	-0.6 (-0.68, -0.52)	-0.04 (-0.12,0.05)	-0.07 (-0.16,0.02)
30-34	-0.54 (-0.62, -0.46)	-0.04 (-0.12,0.03)	-0.08 (-0.16,0.01)
35-39	-0.48 (-0.55, -0.41)	-0.05 (-0.12,0.02)	-0.08 (-0.15,0)
40-44	-0.42 (-0.48, -0.35)	-0.05 (-0.11,0.01)	-0.08 (-0.15, -0.01)
45-49	-0.36 (-0.42, -0.29)	-0.05 (-0.11,0.01)	-0.08 (-0.15, -0.01)
50-54	-0.3 (-0.36, -0.23)	-0.06 (-0.12,0)	-0.08 (-0.15,-0.01)
55-59	-0.23 (-0.3,-0.17)	-0.06 (-0.12,-0.01)	-0.09 (-0.16,-0.01)
60-64	-0.17 (-0.24,-0.1)	-0.07 (-0.12,-0.01)	-0.09 (-0.17,-0.01)
65-69	-0.11 (-0.19,-0.04)	-0.07 (-0.13,-0.01)	-0.09 (-0.18,0)
70-74	-0.05 (-0.13,0.03)	-0.07 (-0.14,-0.01)	-0.09 (-0.19,0)
75-79	0.01 (-0.08,0.1)	-0.08 (-0.14,-0.01)	-0.09 (-0.2,0.01)
80-84	0.07 (-0.03,0.17)	-0.08 (-0.15,-0.01)	-0.1 (-0.21,0.02)
85-89	0.13 (0.03,0.24)	-0.09 (-0.16,-0.01)	-0.1 (-0.23,0.03)
90-94	0.19 (0.08,0.31)	-0.09 (-0.17,-0.01)	-0.1 (-0.24,0.04)
**Period**			
1990-1995	-0.17 (-0.22,-0.11)	0.01 (-0.04,0.06)	-0.02 (-0.08,0.04)
1995-2000	-0.17 (-0.23,-0.12)	-0.11 (-0.16,-0.06)	-0.11 (-0.16,-0.05)
2000-2005	0 (0,0)	0 (0,0)	0 (0,0)
2005-2010	-0.03 (-0.08,0.02)	0.01 (-0.03,0.06)	0.01 (-0.05,0.06)
2010-2015	0.1 (0.05,0.15)	-0.05 (-0.09,0)	-0.05 (-0.11,0)
2015-2020	0.1 (0.05,0.15)	-0.06 (-0.1,-0.02)	-0.07 (-0.13,-0.01)
**Cohort**			
1898-1902	-0.12 (-1.28,1.04)	-0.02 (-0.66,0.63)	0.02 (-1.6,1.65)
1903-1907	-0.05 (-0.5,0.39)	0.06 (-0.2,0.32)	0.09 (-0.51,0.68)
1908-1912	-0.05 (-0.3,0.2)	0.07 (-0.09,0.23)	0.08 (-0.23,0.4)
1913-1917	-0.05 (-0.22,0.12)	0.06 (-0.05,0.17)	0.08 (-0.12,0.27)
1918-1922	-0.06 (-0.19,0.07)	0.05 (-0.04,0.14)	0.06 (-0.08,0.2)
1923-1927	-0.07 (-0.18,0.03)	0.04 (-0.04,0.11)	0.04 (-0.07,0.15)
1928-1932	-0.09 (-0.17,0)	0.01 (-0.06,0.07)	0.01 (-0.08,0.11)
1933-1937	-0.07 (-0.15,0.01)	-0.01 (-0.07,0.05)	-0.01 (-0.09,0.07)
1938-1942	-0.08 (-0.15,0)	-0.04 (-0.09,0.02)	-0.04 (-0.12,0.04)
1943-1947	0 (0,0)	0 (0,0)	0 (0,0)
1948-1952	0.13 (0.07,0.2)	0.08 (0.02,0.13)	0.08 (0.02,0.15)
1953-1957	0.22 (0.15,0.28)	0.07 (0.02,0.13)	0.08 (0.01,0.14)
1958-1962	0.33 (0.27,0.4)	0.08 (0.02,0.14)	0.09 (0.02,0.16)
1963-1967	0.5 (0.43,0.57)	0.16 (0.1,0.23)	0.18 (0.11,0.26)
1968-1972	0.6 (0.52,0.67)	0.13 (0.05,0.2)	0.16 (0.08,0.24)
1973-1977	0.71 (0.62,0.8)	0.08 (-0.02,0.18)	0.11 (0.02,0.21)
1978-1982	0.75 (0.65,0.85)	-0.01 (-0.15,0.12)	0.02 (-0.1,0.15)
1983-1987	0.71 (0.58,0.83)	-0.18 (-0.36,0.01)	-0.13 (-0.29,0.04)
1988-1992	0.64 (0.45,0.83)	-0.33 (-0.64,-0.03)	-0.27 (-0.53,-0.02)
1993-1997	0.75 (0.37,1.13)	-0.34 (-1,0.33)	-0.27 (-0.81,0.26)

Different trends in the net period effect on cervical cancer incidence, mortality rate, and DALYs were observed (**Figure 2**). The incidence increased significantly over the 2005–2019 period. Specifically, the RR of incidence increased by 2.04 times over this period. However, the analysis of the net period effect on cervical cancer mortality and DALYs revealed stable trends.

**Figure 3:**
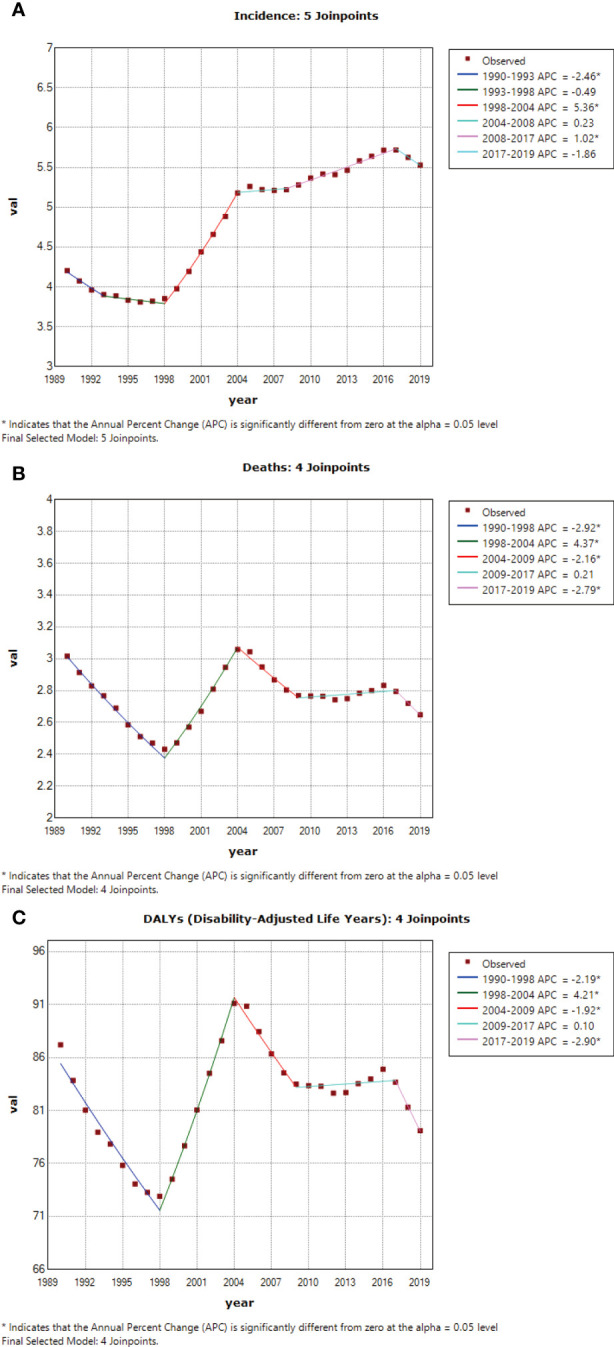
**(A)** Joint point analysis of incidence, **(B)** Joint point analysis of death, **(C)** Joint point analysis of DALY.

The analysis of the cohort effect revealed a substantial increase in cervical cancer incidence from earlier to later birth cohorts (**Figure 2**). From the 1948–1952 birth cohort to the 1993–1997 birth cohort, the RR increased significantly by 85.96%. The cohort effect led to slight increases in the mortality rate and DALYs from earlier (1948-1952) to later (1963-1967) birth cohorts, with increases in the RR of mortality and DALYs of 9.25% and 11.11%, respectively (**Figure 2**) (**Table 4**). The Joint point analysis of incidence, DALYs, and mortality rate were shown in **Figure 3**.

## Discussion

This study showed that the age-standardized cervical cancer incidence increased significantly from 1990 to 2019, whereas the age-standardized mortality rate and DALYs decreased significantly. The APC model showed that the age effect on cervical cancer led to a slight increase in the RR of cervical cancer with advancing age, the incidence increased significantly over the 2005–2020 period, and a substantial increase in the incidence was observed from earlier to later birth cohorts.

Other studies also performed similar analysis in different countries. For example, an Age–Period–Cohort (APC) model from five Population Based Cancer Registries (PBCRs) in India found that a significant age, cohort and period effect was noted in Bangalore, Chennai and Delhi for both breast and cervical cancers (12). Another age-period-cohort analysis suggested that temporal trends in cervical cancer mortality rates could be attributed to period and cohort effects in Lithuania (13).

The incidence of cervical cancer increased from 1990 to 2019. Economic development has increased the cumulative exposure to risk factors, such as, a younger age at first sexual intercourse, unsafe sex, a great number of sexual partners, and human papillomavirus (HPV) infection, and has thus contributed to the increased incidence (14, 15). In Chinese women, the incidence of cervical cancer is unlikely to show a downward trend without a national screening program, so early screening should be emphasized (16).

At present, the design of China’s social medical insurance system is out of touch with the public health service provision system. Basic medical insurance does not cover the cost of women’s health examinations, and the cost of physical examinations still needs to be borne by individuals. The new round of deepening the medical and health system reform started in 2009 decided to include the breast and cervical cancer screening projects in rural areas into major national public service projects (17), and provide free screening services for the two cancers for women of school age in rural areas. The government should fully strive for matching funds, social funds and overall integration funds, establish a fund for “two cancers” screening, include medical insurance coverage and New Cooperative Medical System reimbursement, and benefit all women (including marginalized or disadvantaged women).

Notably, the age-standardized incidence, DALYs, and mortality rate increased significantly from 1998 to 2014 in our study, and a population-based cervical cancer screening study showed that the incidence of cervical cancer increased at an average annual rate of 8.7% from 1989 to 2008 (18). The earlier age of sexual debut in women, increased trends in the rates of multiple sexual partners, extramarital sexual intercourse, and sexual behaviors are likely to increase the incidence of cervical cancer by indirectly increasing the risk of HPV infection (19) and thus contribute further to the increased trends (20). China approved HPV vaccination in 2016, with relatively low coverage (21). The effective control of cervical cancer remains a challenge in China because HPV vaccination is not covered under medical insurance (22).

Persistent infection with a high-risk HPV (hrHPV) strain is a necessary cervical cancer etiology. Compelling evidence has confirmed that vaccination programs targeting the most common hrHPV strains would prevent approximately 87% of cervical cancer cases worldwide (23). There is an urgent need to establish a nationwide comprehensive prevention and treatment system that combines cervical cancer screening with vaccination and is suitable for the Chinese context.

The rate of cervical cancer mortality in China has declined for several reasons. First, economic development has led to an improvement in the overall health status of society and an increase in the average life expectancy. Second, the gradual improvement of China’s women’s health care system has led to an improvement in reproductive health and reduced women’s risk of exposure to cervical cancer-related risk factors (24). Third, the development of science and technology is the most important factor. The diagnosis of cervical cancer has become more accurate and refined with the development of medical technology. The early screening, diagnosis, and treatment of various cervical cancers have improved, thereby greatly improving patients’ survival rates and durations (25).

We observed that the incidence of cervical cancer increased in younger age groups, while the DALYs and mortality rate decreased in older age groups. In line with these findings, a worldwide study of trends in cervical cancer incidence found that younger women in several countries, including China, are greatly affected by increases in incidence (26). Several other studies have shown that the victims of cervical cancer in China are getting younger (27). According to the natural course of HPV-induced cervical carcinogenesis, a persistent HPV infection requires 10–20 years of natural evolution to develop into cervical cancer; therefore, young women should be screened regularly. To reduce the incidence of cervical cancer, early prevention and control measures such as early diagnosis and treatment, sexual hygiene education, and the development of good lifestyle habits for sexually active people are particularly important.

The period effect is usually affected by a combination of historical events and environmental factors. In this study, the incidence of cervical cancer increased significantly over the 2005–2020 period. The cohort effect led to a substantial increase in the cervical cancer incidence from earlier to later birth cohorts. With advances in society, the emergence of early sexual intercourse, multiple sexual partners, unsafe sex have led to an increase in the HPV infection rate, which was shown to be significantly positively correlated with the cervical cancer incidence (28).

Our study provided a comprehensive estimation of cervical cancer burden in China from 1990 to 2019, the data of this study had great public health significance. As well known, China has a vast territory, unbalanced economic development between the east and the west, and uneven distribution of medical resources in urban and rural areas. Therefore, for cities with sufficient medical resources, HPV primary screening or combined HPV detection and cytology screening can be used to improve the accuracy of screening. For the economically underdeveloped rural areas with high incidence of cervical cancer, medical resources are relatively short, and cytology primary screening, or HPV self-sampling detection can be used according to local actual conditions to improve screening coverage.

The APC model is a commonly used model for the analysis of long-term trend of the incidence of diseases (especially cancer) in the population over time (10, 11). Age effects represent the difference in the risk of the outcome associated with different age groups; period effects represent the effect of a combination of historical events and environmental factors; and birth cohort effects represent the features of each generation, including risk factors and exposure to environmental factors in early life.

The APC analysis is an ecological study, thus causal inferences can’t be inferred. In addition, GBD estimates reply on the quality and quantity of the collected data, and inaccuracies in the data may affect our findings. Therefore, the results of this study should be interpreted with caution. Moreover, due to the data of each administrative region in China were not available in GBD database, therefore we cannot analyze it.

## Conclusions

The age-standardized incidence of cervical cancer in China increased from 1990 to 2019, particularly in younger age groups; however, the associated DALYs and mortality rate decreased in older age groups over the same period. In summary, the incidence increased with age, period, and cohort. Early screening for cervical cancer should be emphasized to reduce the disease burden.

## Data availability statement

The original contributions presented in the study are included in the article/supplementary material. Further inquiries can be directed to the corresponding author.

## Author contributions

XS and YC conceived the study. FR and ZS collected and analyzed the data. XS interpreted the results. XS wrote the first draft of the manuscript. XS and YC revised and finalized the manuscript. All authors read and approved the final version of the manuscript.

## Funding

This research was funded by General project of Medical and health Technology Plan of Zhejiang Province (2020381391).

## Acknowledgments

We thank all authors for their contributions to the article.

## Conflict of interest

The authors declare that the research was conducted in the absence of any commercial or financial relationships that could be construed as a potential conflict of interest.

## Publisher’s note

All claims expressed in this article are solely those of the authors and do not necessarily represent those of their affiliated organizations, or those of the publisher, the editors and the reviewers. Any product that may be evaluated in this article, or claim that may be made by its manufacturer, is not guaranteed or endorsed by the publisher.

## References

[1] Plagens-Rotman K Połocka-Molińska M Merks P Gwoździcka-Piotrowska M Kędzia W Jarząbek-Bielecka G. Problems in gynaecologic oncology in girls and young women–an outline of selected issues. Clin Exp Obstet Gynecol (2021) 48(4):795–9. doi: 10.31083/j.ceog4804127

[2] Xiao-Feng Li H-YC Wang Y-Y. Yan-xia sui, guan-jun zhang, xi liu, cervical human papillomavirus infection, genotyping and the relationship with the results of thinprep cytology test in 9174 female physical examinees in xi’an, China. Eur J Gynaecol Oncol (2021) 42(3):537–40. doi: 10.31083/j.ejgo.2021.03.2196

[3] Shrestha AD Neupane D Vedsted P Kallestrup P. Cervical cancer prevalence, incidence and mortality in low and middle income countries: A systematic review. Asian Pac J Cancer Prev (2018) 19(2):319–24. doi: 10.22034/apjcp.2018.19.2.319 PMC5980914 29479954

[4] Arbyn M Weiderpass E Bruni L de Sanjosé S Saraiya M Ferlay J. Estimates of incidence and mortality of cervical cancer in 2018: A worldwide analysis. Lancet Glob Health (2020) 8(2):e191–203. doi: 10.1016/s2214-109x(19)30482-6 PMC7025157 31812369

[5] Li H Jin S Xu H Thomas DB. The decline in the mortality rates of cervical cancer and a plausible explanation in Shandong, China. Int J Epidemiol (2000) 29(3):398–404. doi: 10.1093/ije/29.3.398 10869309

[6] Wang J Bai Z Wang Z Yu C. Comparison of secular trends in cervical cancer mortality in China and the united states: An age-Period-Cohort analysis. Int J Environ Res Public Health (2016) 13(11):1148. doi: 10.3390/ijerph13111148 PMC5129358 27869688

[7] GBD 2019 Diseases and Injuries Collaborators. Global burden of 369 diseases and injuries in 204 countries and territories, 1990-2019: A systematic analysis for the global burden of disease study 2019. Lancet (2020) 396(10258):1204–22. doi: 10.1016/s0140-6736(20)30925-9 PMC7567026 33069326

[8] Kim HJ Fay MP Feuer EJ Midthune DN. Permutation tests for joinpoint regression with applications to cancer rates. Stat Med (2000) 19(3):335–51. doi: 10.1002/(sici)1097-0258(20000215)19:3<335::aid-sim336>3.0.co;2-z 10649300

[9] Clegg LX Hankey BF Tiwari R Feuer EJ Edwards BK. Estimating average annual per cent change in trend analysis. Stat Med (2009) 28(29):3670–82. doi: 10.1002/sim.3733 PMC2843083 19856324

[10] Liu X Yu C Bi Y Zhang ZJ. Trends and age-period-cohort effect on incidence and mortality of prostate cancer from 1990 to 2017 in China. Public Health (2019) 172:70–80. doi: 10.1016/j.puhe.2019.04.016 31220754

[11] Liu X Jiang J Yu C Wang Y Sun Y Tang J. Secular trends in incidence and mortality of bladder cancer in China, 1990-2017: A joinpoint and age-period-cohort analysis. Cancer Epidemiol (2019) 61:95–103. doi: 10.1016/j.canep.2019.05.011 31176961

[12] Sathishkumar K N V Badwe RA Deo SVS Manoharan N Malik R. Trends in breast and cervical cancer in India under national cancer registry programme: An age-Period-Cohort analysis. Cancer Epidemiol (2021) 74:101982. doi: 10.1016/j.canep.2021.101982 34280846

[13] Everatt R Intaitė B. Trends in cervical cancer mortality rates in Lithuania, 1987-2016. Cancer Epidemiol (2018) 57:85–9. doi: 10.1016/j.canep.2018.10.008 30347336

[14] Moore MA Tajima K. Cervical cancer in the asian pacific-epidemiology, screening and treatment. Asian Pac J Cancer Prev (2004) 5(4):349–61. 15546236

[15] Jiang X Tang H Chen T. Epidemiology of gynecologic cancers in China. J Gynecol Oncol (2018) 29(1):e7. doi: 10.3802/jgo.2018.29.e7 29185265 PMC5709533

[16] Zhang S Xu H Zhang L Qiao Y. Cervical cancer: Epidemiology, risk factors and screening. Chin J Cancer Res (2020) 32(6):720–8. doi: 10.21147/j.issn.1000-9604.2020.06.05 PMC7797226 33446995

[17] Han H Wang X Zhu Y Liang Y. Organized breast and cervical cancer screening: Attendance and determinants in rural China. Int J Environ Res Public Health (2022) 19(14):8237. doi: 10.3390/ijerph19148237 35886089 PMC9318997

[18] Hu SY Zheng RS Zhao FH Zhang SW Chen WQ Qiao YL. [Trend analysis of cervical cancer incidence and mortality rates in Chinese women during 1989-2008]. Zhongguo Yi Xue Ke Xue Yuan Xue Bao (2014) 36(2):119–25. doi: 10.3881/j.issn.1000-503X.2014.02.001 24791788

[19] Jiang J Pang H Liu B Nasca PC Zhang B Wu Y. Effects of active, passive, and combined smoking on cervical cancer mortality: A nationwide proportional mortality study in Chinese urban women. Cancer Causes Control (2015) 26(7):983–91. doi: 10.1007/s10552-015-0580-x 25898822

[20] Zhao FH Tiggelaar SM Hu SY Xu LN Hong Y Niyazi M. A multi-center survey of age of sexual debut and sexual behavior in Chinese women: Suggestions for optimal age of human papillomavirus vaccination in China. Cancer Epidemiol (2012) 36(4):384–90. doi: 10.1016/j.canep.2012.01.009 PMC5523958 22377277

[21] Pan XF Li R Pan A Larson H. Human papillomavirus vaccine approval in China: A major step forward but challenges ahead. Lancet Infect Dis (2016) 16(12):1322–3. doi: 10.1016/s1473-3099(16)30450-9 27998583

[22] Yin Y. HPV vaccination in China needs to be more cost-effective. Lancet (2017) 390(10104):1735–6. doi: 10.1016/s0140-6736(17)32606-5 29047442

[23] Bosch FX Lorincz A Muñoz N Meijer CJ Shah KV. The causal relation between human papillomavirus and cervical cancer. J Clin Pathol (2002) 55(4):244–65. doi: 10.1136/jcp.55.4.244 PMC1769629 11919208

[24] Di J Rutherford S Chu C. Review of the cervical cancer burden and population-based cervical cancer screening in China. Asian Pac J Cancer Prev (2015) 16(17):7401–7. doi: 10.7314/apjcp.2015.16.17.7401 26625735

[25] Wang B He M Chao A Engelgau MM Saraiya M Wang L. Cervical cancer screening among adult women in China, 2010. Oncologist (2015) 20(6):627–34. doi: 10.1634/theoncologist.2014-0303 PMC4571778 25956407

[26] Vaccarella S Lortet-Tieulent J Plummer M Franceschi S Bray F. Worldwide trends in cervical cancer incidence: Impact of screening against changes in disease risk factors. Eur J Cancer (2013) 49(15):3262–73. doi: 10.1016/j.ejca.2013.04.024 23751569

[27] Chen W Sun H Molijn A Zeng L Kang L Jenkins D. The variable characteristics of human papillomavirus in squamous cell carcinoma and adenocarcinoma of cervix in China. J Low Genit Tract Dis (2018) 22(4):355–61. doi: 10.1097/lgt.0000000000000408 30074955

[28] Dong Z Hu R Du Y Tan L Li L Du J. Immunodiagnosis and immunotherapeutics based on human papillomavirus for HPV-induced cancers. Front Immunol (2020) 11:586796. doi: 10.3389/fimmu.2020.586796 33488587 PMC7820759

